# Heidenhain Variant of Creutzfeldt-Jakob Disease: A Case Report

**DOI:** 10.7759/cureus.67848

**Published:** 2024-08-26

**Authors:** Nikolina Madjer, Rahul Shaju, Colin Vipond, Andrew MacDougall, Pavan Murty

**Affiliations:** 1 Internal Medicine, Advocate Lutheran General Hospital, Park Ridge, USA; 2 Neurology, Advocate Lutheran General Hospital, Park Ridge, USA

**Keywords:** cortical ribboning, ocular symptoms, prion diseases, creutzfeld-jakob disease, heidenhain variant

## Abstract

Creutzfeldt-Jakob disease (CJD) is a rare, rapidly progressive, fatal neurodegenerative disorder caused by an accumulation of protein-containing particles called prions in the central nervous system. The Heidenhain variant (HvCJD) is a rare subtype of CJD that presents with predominantly visual symptoms at onset. The patient presented in this case had several weeks of visual symptoms prior to hospital admission. Due to the rare nature of this disease, this patient underwent a substantial and invasive workup of her symptoms that eventually led to her being diagnosed with an incurable disease. The aim of this report is to highlight the clinical presentation and diagnostic evaluation of a patient suffering from HvCJD, with a focus on the initial presentation of progressive vision loss prior to the onset of cognitive impairment.

## Introduction

Creutzfeldt-Jakob disease (CJD) occurs in one in one million people worldwide annually with most cases (>90%) being sporadic [1]. Sporadic CJD (sCJD) is caused by a spontaneous conversion of a normal cellular prion protein (PrP^C^) into an abnormal isomer (PrP^Sc^) [2]. sCJD is further classified into six different variants based on the genotype of the PNRP gene. The gene may be homozygous or heterozygous for the presence of methionine or valine on codon 129 [3]. The molecular mechanism behind the origin of PrP^Sc^ is unknown; however, the presence of PrP^C^ is required for PrP^Sc^ to form [4]. Current molecular models theorize that PrP^Sc^ plays a role in converting more PrP^C^ to its abnormal form [3]. PrP^Sc^ is resistant to protease degradation, and its eventual accumulation in the cytoplasm of neurons triggers cellular apoptosis. 

CJD is clinically characterized by rapidly progressive dementia and often myoclonus and cerebellar dysfunction. Other symptoms of classic CJD that have been documented include memory impairment, ataxia, and disorientation [3]. Several clinical subtypes of CJD have been described based on symptomatology including Oppenheimer-Brownell CJD which solely presents with ataxia [3]. Heidenhain variant (HvCJD), however, was first described by Adolf Heidenhain in 1929 [5]. HvCJD accounts for approximately 5% of the total number of CJD cases [6]. It is clinically characterized by visual disturbances at onset including blurred vision, impaired perception of colors and structures, visual hallucinations, vision loss, and visual anosognosia without evidence of ocular pathology [3,5]. As visual symptoms develop prior to cognitive decline, patients often undergo ophthalmologic evaluation prior to neurologic evaluation [6]. 

We describe a patient with preceding progressive vision loss attributed to cataracts, who presented to the hospital following a fall with resultant traumatic brain injury and subsequently experienced cognitive decline during hospitalization leading to an ultimate diagnosis of HvCJD. 

## Case presentation

**Figure 1:**
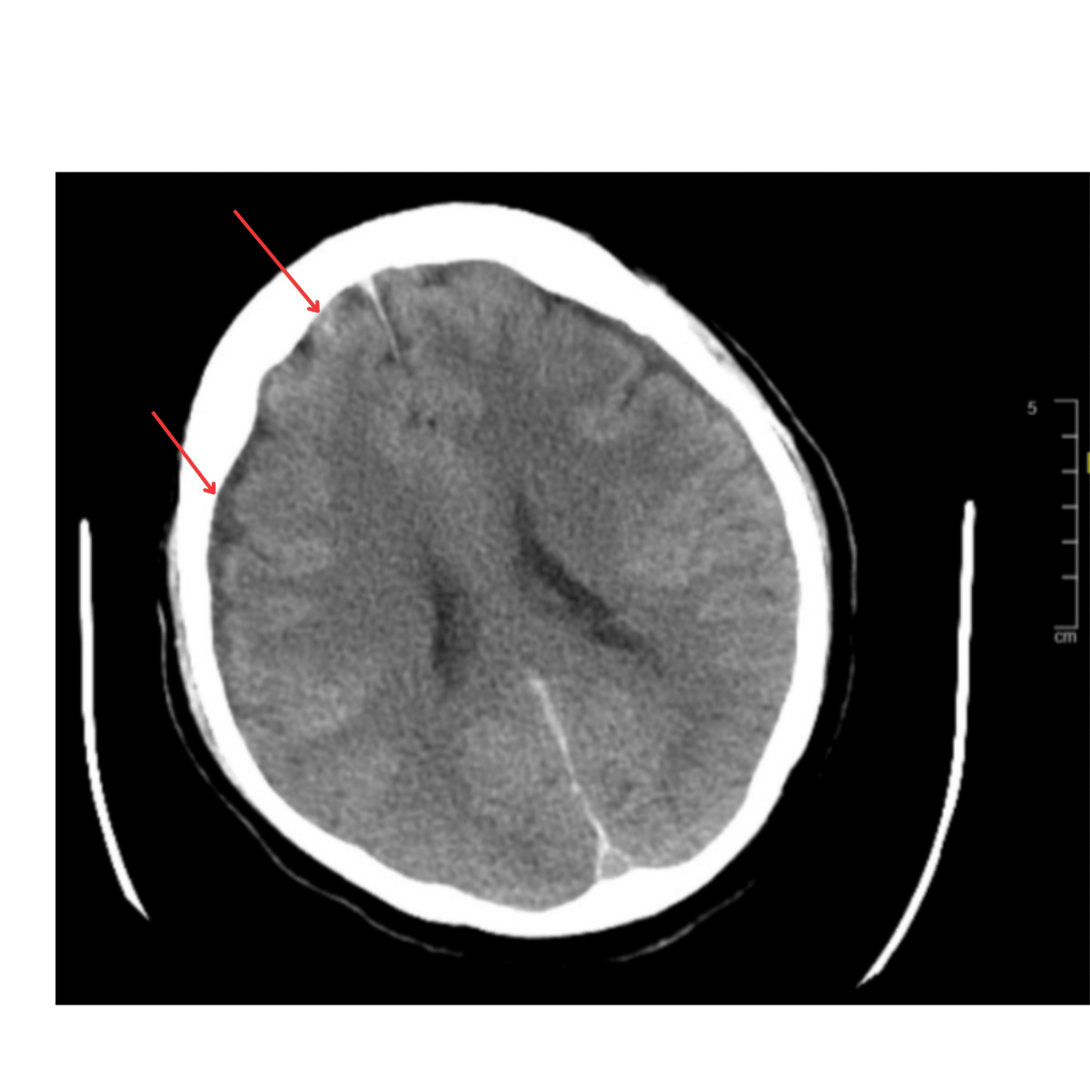
CT head without contrast CT: Computed tomography The top left arrow is pointing to the minor right frontal subarachnoid hemorrhage. The bottom left arrow is pointing to a small hemorrhage in the right frontoparietal sulci

A 72-year-old female with a past medical history significant for hypertension and chronic kidney disease presented as a trauma transfer from another hospital for evaluation following a fall. The patient's family had reportedly found her at home on the floor near a staircase confused and with multiple bruises. Initial imaging was significant for multiple axial and appendicular bone fractures, a small right frontal subarachnoid hemorrhage, and a small hemorrhage in the right frontoparietal region on computed tomography (CT) of the head (Figure 1). 

Collateral history was obtained from the patient’s son. A few months ago, the patient was fully functional and lived independently. She was an avid cyclist, often cycling 10 miles a day. She subsequently developed blurred vision several months prior and was evaluated by several ophthalmologists who diagnosed her with cataracts. She then underwent surgical treatment two months prior to this admission. Despite this, her vision loss progressed, with suspected lens dislocation as the underlying cause. She also developed vertigo and unsteady gait. She presented to an emergency room a month prior and underwent CT of the head which was reported as normal. She did not have cognitive deficits prior to this hospitalization. 

On initial neurologic evaluation, the patient was awake but poorly attentive and unable to provide history. She answered limited simple questions and followed basic commands with encouragement. She had a normal pupillary light reflex, intact extraocular movements, and symmetric antigravity movement of extremities. She had significant vision loss with inability to count fingers and identify objects or colors though was unaware of her visual deficit. She underwent serial CT imaging of the head which demonstrated stable subarachnoid hemorrhage though her cognitive symptoms worsened. 

**Figure 2:**
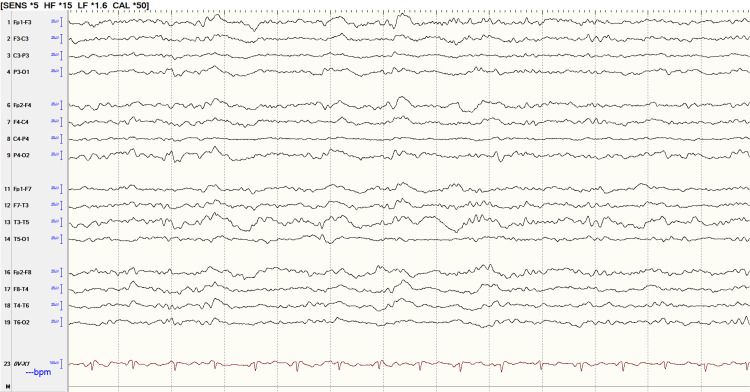
Electroencephalogram (EEG)

**Figure 3:**
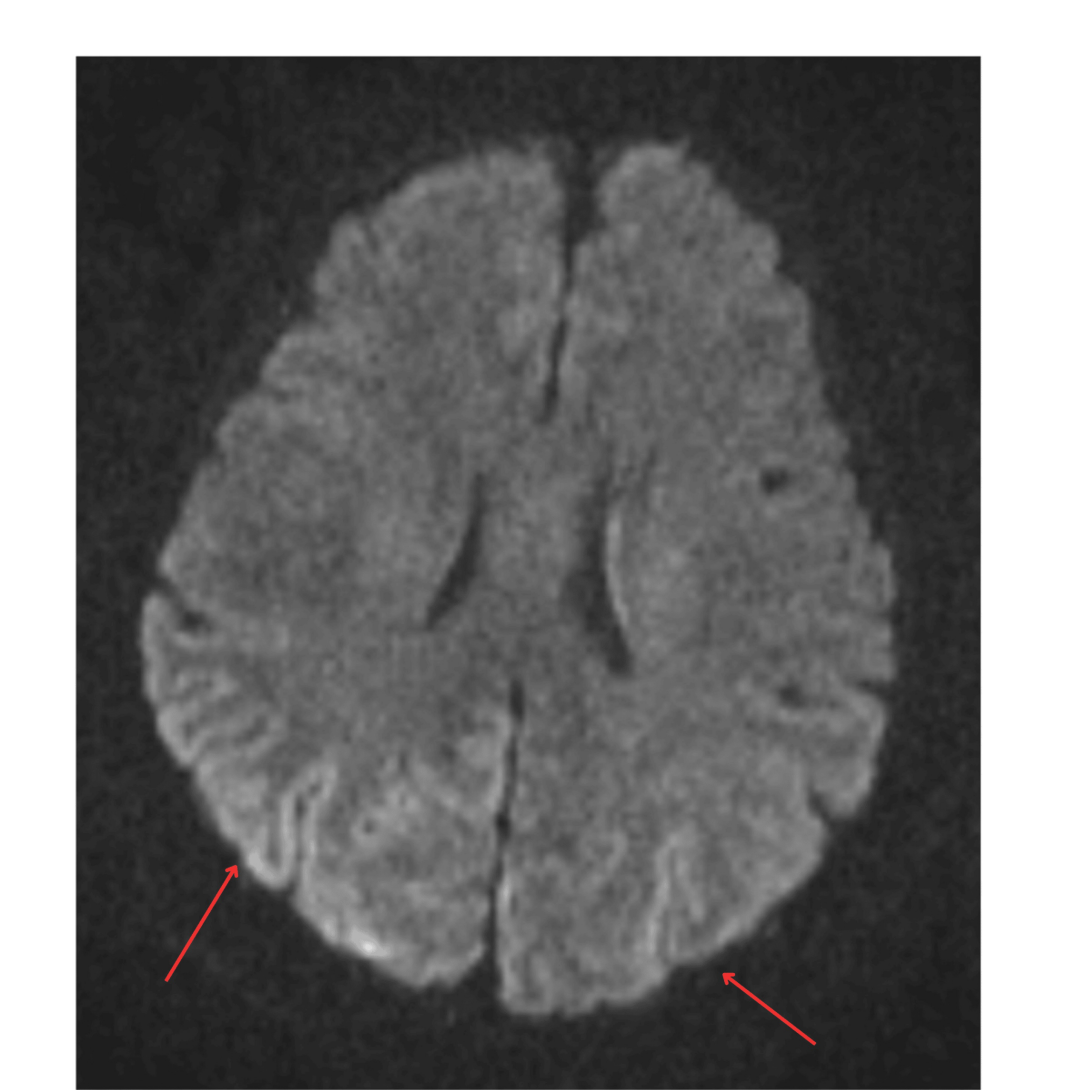
MRI brain, diffusion-weighted imaging MRI: Magnetic resonance imaging Cortical diffusion restriction of both cerebral hemispheres posterolaterally indicated by the two bilateral arrows. Limited study due to motion artifact

Further neurologic testing was obtained in the hospital including routine electroencephalogram (EEG) and magnetic resonance imaging (MRI) of the brain. EEG was abnormal with nonspecific findings including moderate diffuse background slowing and generalized rhythmic delta activity (Figure 2). MRI was abnormal with restricted diffusion in the bilateral parieto-occipital cortex (cortical ribboning (Figure 3). 

Based on MRI findings, CJD was considered among other diagnoses, and a lumbar puncture was performed for further evaluation. Initial cerebrospinal fluid (CSF) evaluation was notable for an absence of pleocytosis and an elevated protein (Table 1). Other considerations included autoimmune or paraneoplastic encephalitis, and the patient was treated with five days of high-dose intravenous methylprednisolone (1000 mg per day). There was no subsequent improvement in mental status. 

**Table 1:** Multiple sclerosis panel, CSF CSF: Cerebrospinal fluid

Multiple sclerosis panel, CSF	Patient value	Reference range
Protein	96	15-45 mg/dL
Albumin	61	0-35 mg/dL
Immunoglobulin G	8.8	0.9-6.1 mg/dL
CSF IgG index	0.49	<0.76
Immunoglobulin G, CSF synthesis rate	<1.0	0.0-8.0 mg/day
IgG/albumin Index	0.14	0.09-0.25
CSF electrophoresis interpretation	Negative for oligoclonal bands. This would not be supportive of multiple sclerosis	

CSF evaluation included testing for infectious, autoimmune, paraneoplastic, and neoplastic conditions. Infectious workup included a CSF bacterial culture with no growth and a negative polymerase chain reaction panel testing for viruses, bacteria, and fungi associated with meningitis and encephalitis (Table 2). IgG index was not elevated, and there was an absence of oligoclonal bands (Table 1). A panel testing for antibodies associated with autoimmune and paraneoplastic encephalitis was negative. Cytology showed no malignant cells (Table 3). Serum HIV testing was negative. 

**Table 2:** Rapid meningitis/encephalitis CSF CSF: Cerebrospinal fluid

Rapid meningitis/encephalitis CSF	Patient value	Reference range
*Cryptococcus neoformans*/*gattii*	Not detected	Not detected
Cytomegalovirus (CMV)	Not detected	Not detected
Enterovirus (types A-D)	Not detected	Not detected
Haemophilus influenzae	Not detected	Not detected
Herpes simplex virus type 1	Not detected	Not detected
Herpes simplex virus type 2	Not detected	Not detected
Human herpesvirus 6	Not detected	Not detected
Human *parechovirus*	Not detected	Not detected
Listeria monocytogenes	Not detected	Not detected
Neisseria meningitidis	Not detected	Not detected
Streptococcus agalactiae	Not detected	Not detected
Varicella zoster virus (VZV)	Not detected	Not detected

**Table 3:** Cerebrospinal fluid (CSF) cell count and differential This table highlights the presence of xanthochromia and elevated red blood cells (RBCs) likely from the trauma of the lumbar puncture itself

Cerebrospinal fluid cell count and differential	Patient value	Reference range
CSF color	Yellow	
CSF clarity	Clear	
Total nucleated cells	1	0-10/mcL
RBC	562	<1/mcL
Xanthochromia	Present	Absent

During hospitalization, the patient experienced progressive neurologic decline including development of aphasia, decreased level of consciousness, motor weakness, and spasticity. She also had an absent blink-to-threat reflex. 

CSF testing was subsequently found to be positive for real-time quaking-induced conversion (RT-QuIC) (Table 4). Based on these results and MRI findings, the patient was diagnosed with HvCJD. Neurology and palliative care teams discussed the diagnosis with the patient’s family who ultimately pursued hospice care given her continued neurologic decline. The patient expired 30 days following initial presentation and six days following positive CSF RT-QuIC results. 

**Table 4:** Creutzfeldt-Jakob disease, 14-3-3 protein, CSF RT-QuIC: Real-time quaking-induced conversion This table highlights the positive RT-QuIC result along with an extremely elevated T-tau protein value

Creutzfeldt-Jakob disease, 14-3-3 protein, CSF	Patient value	Reference range
RT-QuIC	Positive	Negative
T-tau protein	>20000 pg/mL	0-1149 pg/mL
14-3-3	Test not performed	<30-1999 AU/mL

## Discussion

HvCJD is a rare variant (~5%) of an already extremely rare disease, with CJD occurring in one in one million individuals [6]. This subtype has a unique presentation compared to typical CJD given the isolated visual symptoms at onset. Blurred vision that leads to the avoidance of daily activities, such as reading or watching television, may be the only initial symptom [5]. Due to visual symptoms, up to 77% of patients see ophthalmology as their first medical point of contact [7]. Our patient developed blurred vision as her initial symptom and sought consultation from multiple ophthalmologists. This resulted in multiple ophthalmologic diagnoses that led to an invasive surgical procedure, which is unfortunately not uncommon in other reported cases of this disease subtype [6]. 

Progressive visual disturbances in patients with HvCJD include impaired perception of color and structures, visual hallucinations, vision loss, and visual anosognosia [5]. Examination of our patient’s vision was limited by her mental status though she was noted to have cortical blindness and visual anosognosia given her unawareness of her visual deficit. The patient subsequently developed more typical symptoms including progressive cognitive and cerebellar deficits. Our patient reportedly had symptoms of vertigo and unsteady gait prior to admission though these were not directly observed during her hospitalization. 

Early isolated visual symptoms can be explained by the pronounced involvement of the parietal and occipital lobe as seen on MRI [5], in contrast to the more typically involved regions with CJD including the superior frontal gyrus, superior parietal lobule, cingulate gyrus, and insular cortex. Our patient’s MRI demonstrated cortical ribboning preferentially involving the bilateral parieto-occipital lobes. MRI has 95% sensitivity and 93% specificity rates in diagnosing HvCJD [8]. CSF evaluation shows similar findings to typical CJD including nonspecific elevation of protein, the presence of 14-3-3 protein, and positive RT-QuIC, the latter of which was seen in our patient [6]. RT-QuIC is 80%-90% sensitive and 100% specific for CJD [8]. 

From symptom onset to death, the clinical deterioration in cases of this variant can be rapid. In one case study, duration of disease in the HvCJD as compared to other cases of CJD was characterized by a significantly shorter course. The disease duration of the HvCJD averaged 5.7 months with a range of 2.3-14.1 months as compared to 7.5 months with a range of 1.3-32.4 months in other cases [5]. This may be due to initial nonspecific visual symptoms and delayed development of other neurologic symptoms until later in disease course. Our patient’s cognitive symptoms were only evident following traumatic brain injury, and she had rapid cognitive decline during hospitalization. 

Earlier neurologic evaluation in our patient, specifically MRI brain, at onset of symptoms may have resulted in an earlier diagnosis of HvCJD and avoidance of alternative ophthalmologic diagnoses. This could have also avoided an invasive surgical procedure with the potential for contaminated surgical instruments. 

## Conclusions

Early ocular symptoms of unclear etiology led to this patient's traumatic fall. She subsequently underwent extensive testing, culminating in an invasive surgical procedure, before ultimately being diagnosed with HvCJD. As a result of her injuries, she was able to undergo appropriate testing including an MRI brain and a spinal tap that eventually revealed a positive RT-QuIC. The aim of this case report was to highlight the importance of increased clinical suspicion and early recognition of HvCJD. This allows for further and timely diagnostic evaluation to potentially spare patients from traumatic injuries and avoids invasive, futile procedures that put the patient and the healthcare professionals caring for them at risk of exposure. 
